# Rapid testing on the effect of cracks on solar cells output power performance and thermal operation

**DOI:** 10.1038/s41598-022-16546-z

**Published:** 2022-07-16

**Authors:** Mahmoud Dhimish, Yihua Hu

**Affiliations:** Department of Electronic Engineering, University of York, Heslington, York, YO10 5DD UK

**Keywords:** Solar cells, Engineering

## Abstract

This work investigates the impact of cracks and fractural defects in solar cells and their cause for output power losses and the development of hotspots. First, an electroluminescence (EL) imaging setup was utilized to test ten solar cells samples with differing crack sizes, varying from 1 to 58%. Our results confirm that minor cracks have no considerable effect upon solar cell output, and they develop no hotspots. However, larger cracks can lead to drastic decreases in the output power, close to − 60%. Furthermore, as the crack area increased, there was a further increase in the cell's temperature under standard test conditions. On the contrary, no hotspots were found for the solar cells affected by significant creak areas (crack percentage > 46%) because there were insufficient areas to develop a hotspot. Last, a comparative analysis with solar cells affected by potential induced degradation (PID) was made. We found a strong relationship in the output power losses, and the PID test critically impacted the cells by developing localized hotspots at a temperature level close to 50 °C.

## Introduction

In recent years, cracks in solar cells have become an important issue for the photovoltaic (PV) industry, researchers, and policymakers, as cracks can impact the service life of PV modules and degrade their performance over time^1,2^. Often cracks are named microcracks or µcrack, and all typically indicate a fracture in the solar cells in the range of mm to as small as in micrometres. Both terms usually suggest the same type of cracks where partially fully isolated areas are developed in the solar cells mainly due to mechanical or thermal stresses^3,4^. This stress can result from manufacturing, transportation phase to the PV site, installation process, or heavy snow and physical damage to the modules. Optimizing these processes can reduce cell cracking; cracks during production are unavoidable.

The crack issue in solar cells becomes worse as the thickness of the wafer is being reduced^5^. This is the case because the reduced thickness makes it easier to develop extra mechanical stresses in the cells when assembled into a full-scale PV module. Often, this will cause cracks in the cells and lead to up to 2.5% power degradation in 60-cell PV modules if they do not insulate cell areas. In a relevant study^6^, cracks have been proven to impact the surface structure of the solar cells and extend to damage the fingers and busbars. This would lead to disconnecting cell areas and reducing the maximum generated current. In the recent work by^7,8^, they have shown that solar cell cracks can not only isolate parts of the cells but also, and due to the nature of the cracks themselves, they can develop a localized increase in the temperature, resulting in what is commonly known by "solar cell hotspots".

The mitigation of solar cell cracks has not been yet discovered. However, as cracks lead to hotspots, there were some attempts to mitigate hot spotted solar cells by utilizing a power electronics device to regulate the current into the affected cells^9–12^. These techniques work under the same principle by adding a switching element with high frequency to control the current in the modules and do not affect the interconnection between the module and the power converter. These techniques were approved effective, and they can increase the PV modules output power.

The PV modules are usually connected in series for grid-connected PV systems to build up the voltage output, and the modules frames are grounded for safety purposes^13,14^. A high electric potential difference between the cells and the module farm may be induced in the modules, typically at the PV string level. This phenomenon will result in a leakage current flow from the module frame to the solar cells, which results in a potential induced degradation (PID)^15–17^. Therefore, solar cell cracking and PID are different; however, both lead to a drop in the output power of the modules.

**Figure 1:**
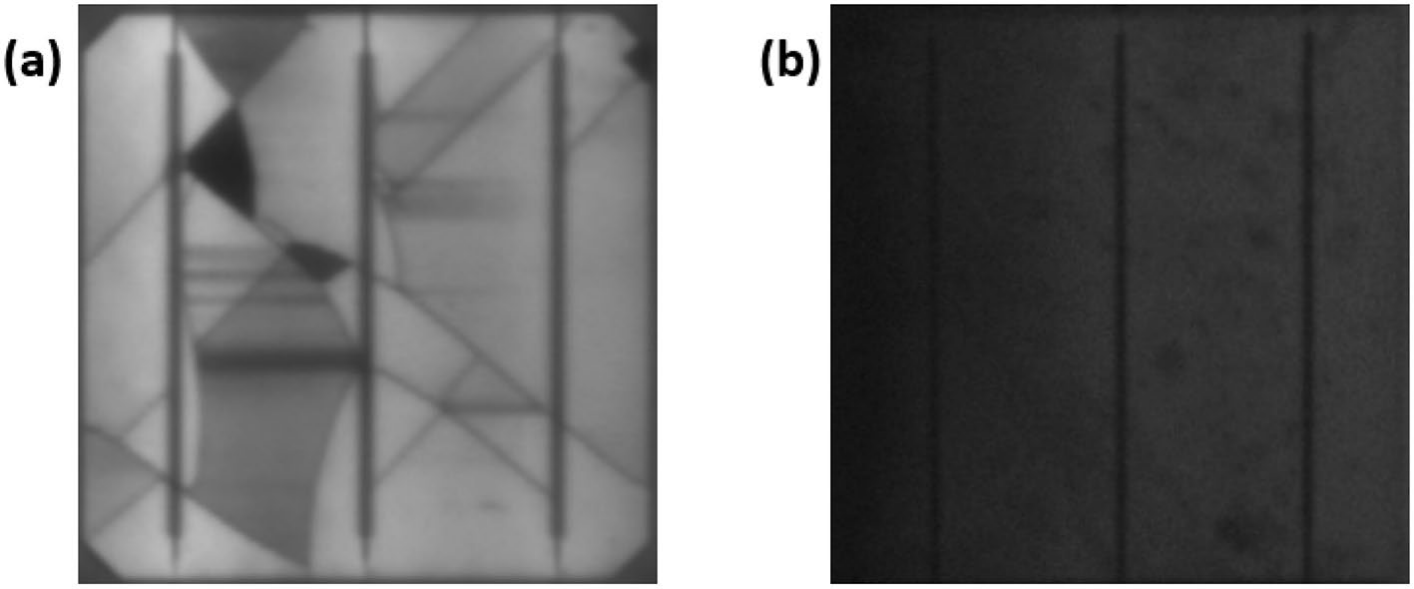
EL image of solar cells (**a**) Affected by cracks and structural defects, (**b**) affected by PID.

Cracks are often invisible to the bare eye; the current standard cracks detection method uses Electroluminescence (EL) imaging^18–20^. In Fig. 1, the EL image of two different solar cells is presented. Here we show the difference between the EL image when a solar cell is affected by cracking and structural defects (Fig. 1a) and when the cell is affected by PID (Fig. 1b). There has been a limited explanation of the behaviour of these cracks on the actual degradation of the output power and their correlation to the presence of hotspots. In addition, to date, there is a lack of understanding of whether all types (or crack percentages) can lead to a significant drop in the output power generation of solar cells.

To date, there is still a gap of knowledge in understanding the impact of cracks on solar cell performance, particularly those exposed in the field under different environmental conditions. Usually, and as explained in multiple previous studies^21–23^, cracks can degrade the PV output power under controlled indoor testing; these various studies, however, do not consider the influence of the size of the cracks and the correlation between the cracks and their thermal impact on the PV modules. In addition, some other recently published work^24,25^ has shown that PV cracks can influence the electrical parameters of the PV modules, while they did not precisely evidence whether the cracks purely cause this degradation in the module, as PV modules exposed in the filed can be affected by other degradation mechanisms such as potential-induced degradation^26^, bypass diodes failure, hotspots, or temperature-induced degradation (TID)^27^.

Considering these research gaps of knowledge, the main contributions of this paper are to provide an understanding of how the output power degradation in solar cells is affected by different sizes of cracks. In addition, the correlation between solar cell cracks and the development of hotspots will also be discussed. Our last contribution is to correlate PID vs cracked solar cells' power losses and resemble their thermal performance.

This paper is organized as follows: “Materials and methods” section comprises the sample preparation and the description of the EL and solar simulator testing setup. “Results and discussion” section demonstrates the results of the tested solar cell samples, including output power measurements and thermal cycling, and “Conclusions” section presents the paper's conclusions.

## Materials and methods

This work explores crystalline silicon (c-Si)-based solar cells affected by different sizes of cracks. The studies cells are made of three busbars, and as provided by the manufacturer datasheet, under standard testing conditions (STC), each cell has an open-circuit voltage ${V}_{oc}$ of 0.61 V, short circuit current density ${J}_{sc}$ of 38.8 mA/cm^2^, and peak power 4.72 W.

**Figure 2:**
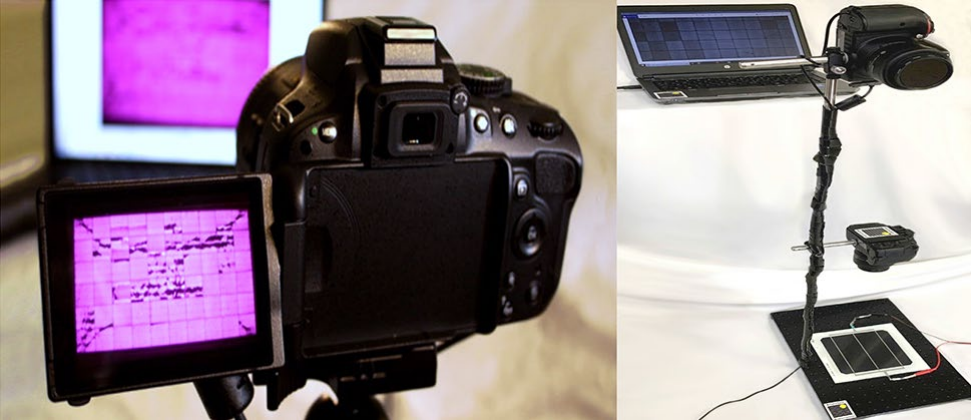
EL imaging setup.

To capture the EL images of the examined solar cell samples, we have used the EL imaging setup shown in Fig. 2. The EL comprise a digital camera with a resolution of $6k \times 4k$ pixels. The used camera lens is 18–55 mm, and the solar cell samples were connected with a power supply for biasing purposes under short circuit conditions.

**Figure 3:**
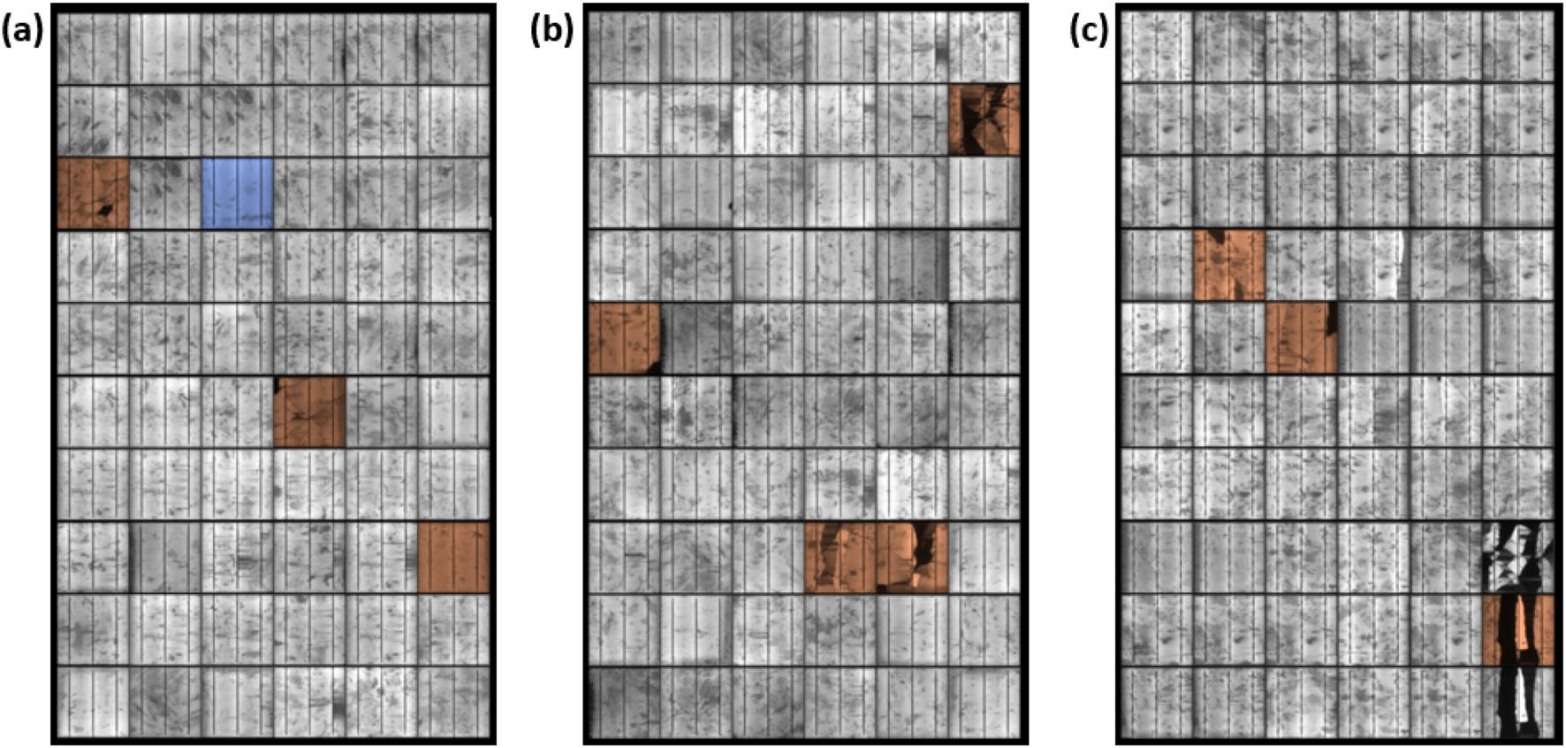
Original EL image for the PV modules before the dismounting process (**a**) PV module #1, (**b**) PV module #2, (**c**) PV module #3.

The tested solar cell samples categorizing different crack shapes on the distribution and structural defects. The EL images of the tested cells are shown in Table 1. The crack size ranges from 1 to 58%. The percentage of the crack was computed by subtracting a cracked vs crack-free image; this was performed using MATLAB script. The solar cell samples were deliberately dismounted from an original three different PV modules; this process was handled by Solar UK Ltd, an international PV recycling company located in the UK. The EL image of the PV modules before the dismounting process had to happen is shown in Fig. 3. There is no difference in the crack size and orientation before and after the dismounting process was completed. The PV modules examined in this work were exposed to outdoor conditions; therefore, we cannot precisely define the source of the cracks (i.e., caused during the PV installation phase, rapid damage due to hailstorm, snow, etc.).

**Table 1:**
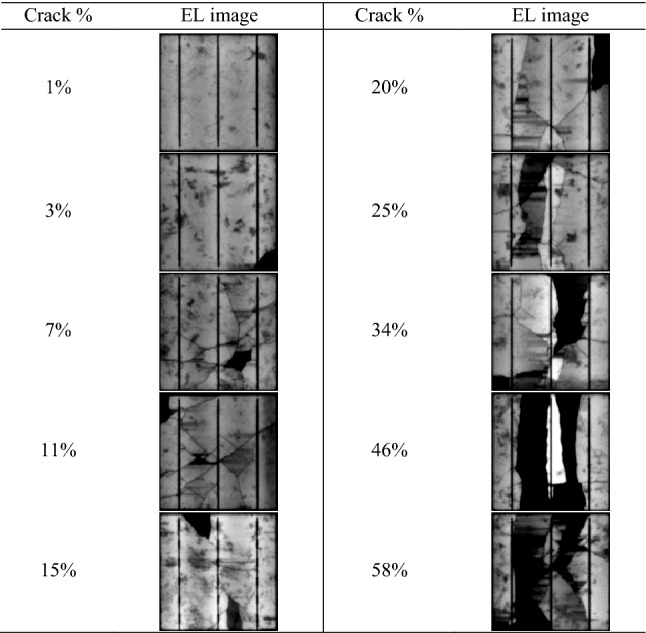
Crack size and EL image of the examined solar cell samples.

**Figure 4:**
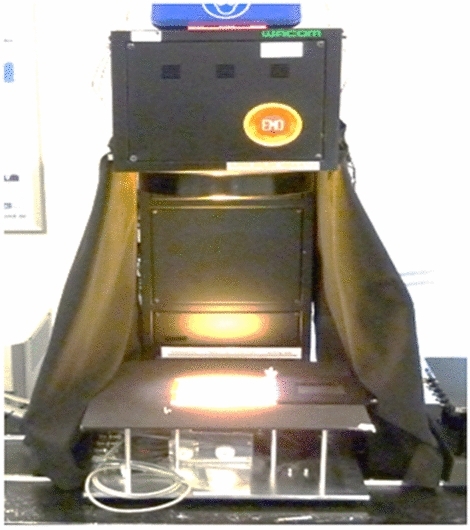
PVcomB solar simulator.

The solar simulator tested the performance of the cells, and the output power vs irradiance was taken at each analysed condition. The class A + solar simulator, shown in Fig. 4, has been used to facilitate the testing of the examined solar cell samples. This instrument has a maximum illumination area of $155 \times 155$ mm^2^, a homogeneity of illumination ± 2 W/m^2^ at irradiance intensity range 0–1000 W/m^2^. In addition, the solar cell temperature can be controlled from 15 to 200 °C via the PVcomB software to maintain a specific temperature level during the experiments. A thermal camera is placed on the back sheet of the solar simulator (solar cell holder) to aid the thermal measurements of the examined samples. The camera has a thermal sensitivity of ± 0.1 °C.

## Results and discussion

This section presents our empirical results of the output power measurements, thermal cycling, and PID testing for the examined solar cell samples.

### Output power measurements

**Figure 5:**
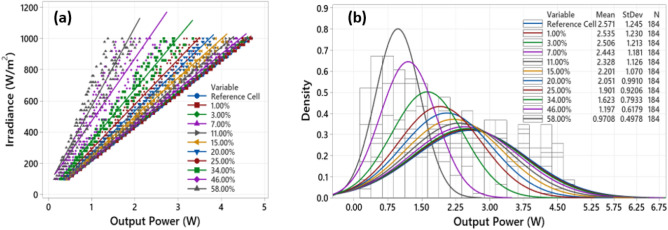
(**a**) Output power vs irradiance for the examined solar cells, (**b**) Density and the calculated parameters of the collected data; N = 184 represents the number of samples of every experiment.

The tested solar cell samples, including the reference cell, were all subjected to solar illumination under varying irradiance, 100–1000 W/m^2^, the temperature of the cells was maintained at STC conditions, 25 °C. The output power measurements taken from the solar simulator is shown in Fig. 5a. The step-change in the irradiance level is 20 W/m^2^, and for precision targets, we took four different measurements for every irradiance level. Next, we have distributed the measured output power as in histogram (Fig. 5b), and a normal distribution function was selected to observe the mean and the standard deviation (StDev). In this figure, the y-axis denotes the density, which corresponds to the relationship between the observations/measurements and their probability of occurrence. Hence, 0.9 resembles a high probability of occurrence than 0.1, which corresponds to a low probability of occurrence. Using this parameter in the y-axis helps find the rectified mean value of the output power of each tested solar cell, therefore, eliminating any errors in the measurements.

As can be observed in Fig. 5b, there is a significant correlation between the output power of the reference cell and the solar cells affected by 1%, 3%, 7% and 11% crack percentages. The mean output power is approximately equal to the reference cell, 2.571 W. In contrast, we observe a significant decay in the mean of the output power while the crack percentage in the solar cell increases. For example, the solar cell affected by 20% has a mean output power of 2.051 W, compared with 0.9708 W identified from the last solar cell sample with a crack percentage of 58%.

In addition, as the crack percentage increase in the solar cell, it is anticipated that the standard deviation of the output power measurements decreases. A low standard deviation indicated that the values are clustered close to the mean. Hence, even though the irradiance increased while experimenting, there were no significant changes in the output power of the critically cracked solar cells (i.e., 46% and 58% crack percentages).

**Figure 6:**
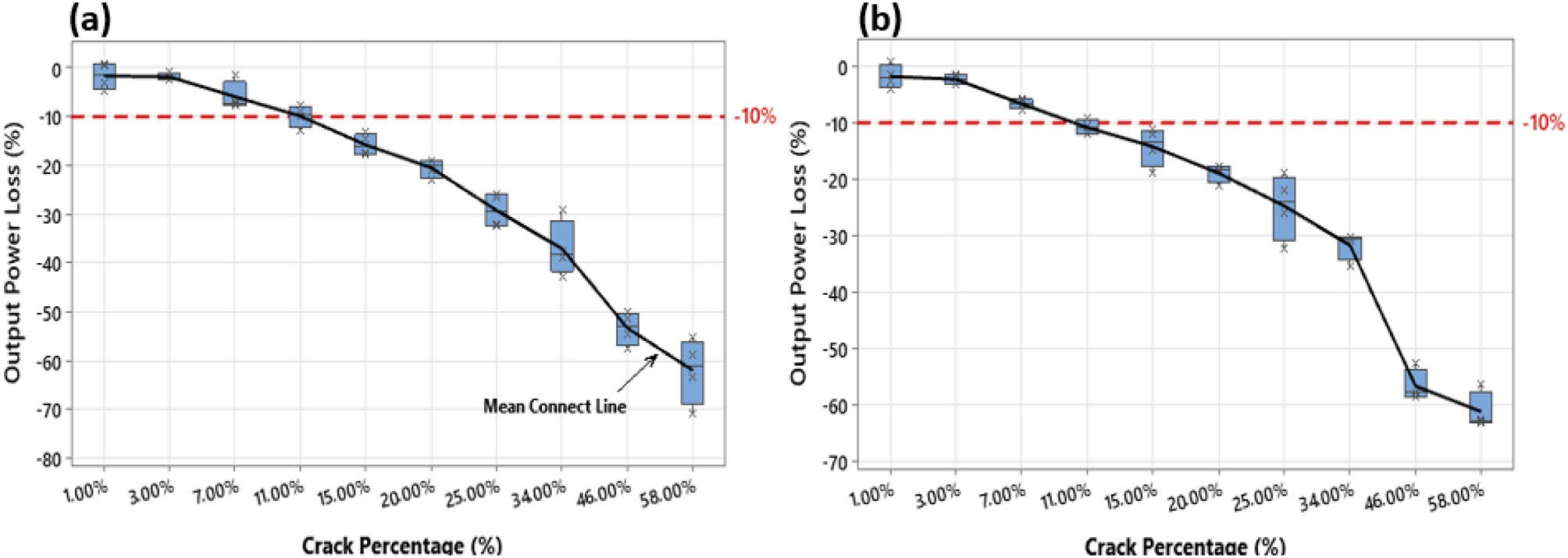
Calculation of the output power loss for the tested cracked solar cell samples (**a**) at 1 Sun, (**b**) at 0.5 Sun.

The output power loss is calculated using (1), where $P_{ref}$ is the output measured power of the reference healthy solar cell, the value of $P_{ref}$ is taken from the manufacturer datasheet, and the $P_{sample}$ is the output measured power for the selected solar cell samples. The results of the output power loss at 1 Sun (1000 W/m^2^) and 0.5 Sun (500 W/m^2^) are presented in Fig. 6.

1 $$ Output \,Power\,Loss\, \left( \% \right) = \frac{{P_{ref} - P_{sample} }}{{P_{ref} }} \times 100 $$

Our baseline to consider whether the crack percentage has a significant loss in the output power is confined at − 10%; this value was selected because:

1. We compared the results of the output power of the cracked solar cell with the reference cell that has been already operated in the field. Consequently, there should be some additional losses/degradation in the output power anyways.
2. We applied the smallest standard deviation ratio of 10% below or above the mean. This threshold will guarantee all measurements are included in the analysis.

According to Fig. 6a, the solar cells with crack percentage below 15% are above the -10% baseline. This result suggests that the output power losses for the solar cells with crack percentages of 1%, 3%, 7%, and 11% is insignificant. We confirm the same outcome while testing with the solar samples at 0.5 Sun, as shown in Fig. 6b. Accordingly, these results enable us to understand that not all cracks in solar cells could induce output power losses. Small cracks, i.e., below 10%, unlikely influence the output power generation and are relatively equivalent to non-cracked cells. In a comparative evaluation, the output losses (or degradation) are likely to transpire due to other predicaments such as encapsulation, arcing-faults, or PID.

### Thermal performance and hotspots development

The thermal images of the solar cells, shown in Table 2, have been taken under STC conditions. These images have been captured approximately 2-min when applying the illumination to make sure that the hotspot develops, if any.

**Table 2:**
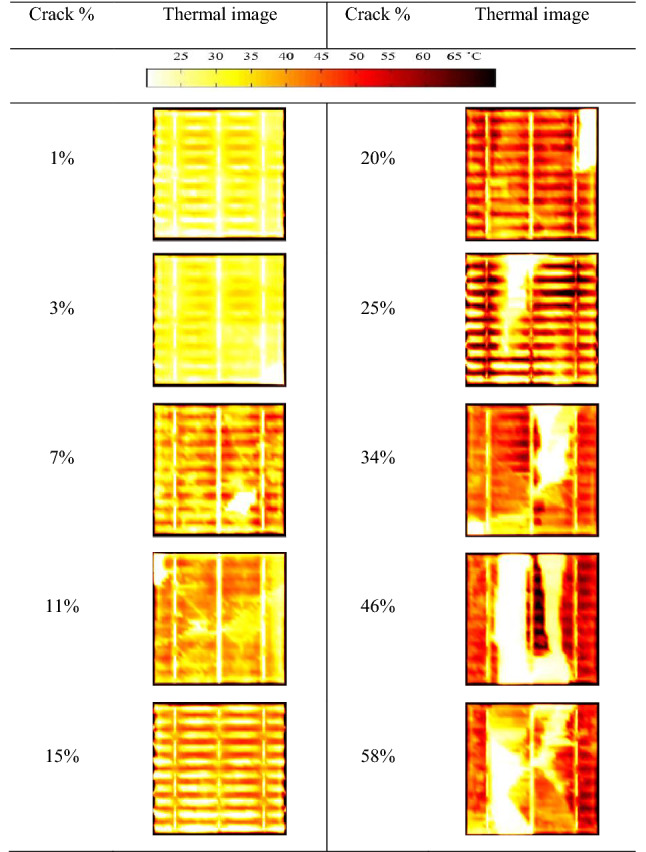
Thermal images of the examined solar cell samples under STC conditions.

Even though the cooling mode of the solar illuminator was set up at 25°, the solar cells have not been left under illumination for a long time to ensure that no rapid increase in the temperature occurs. In addition, following this procedure will guarantee that the temperature increase in some of the tested solar cells results from the cracks, not because of the rapid illumination of the sun simulator.

**Figure 7:**
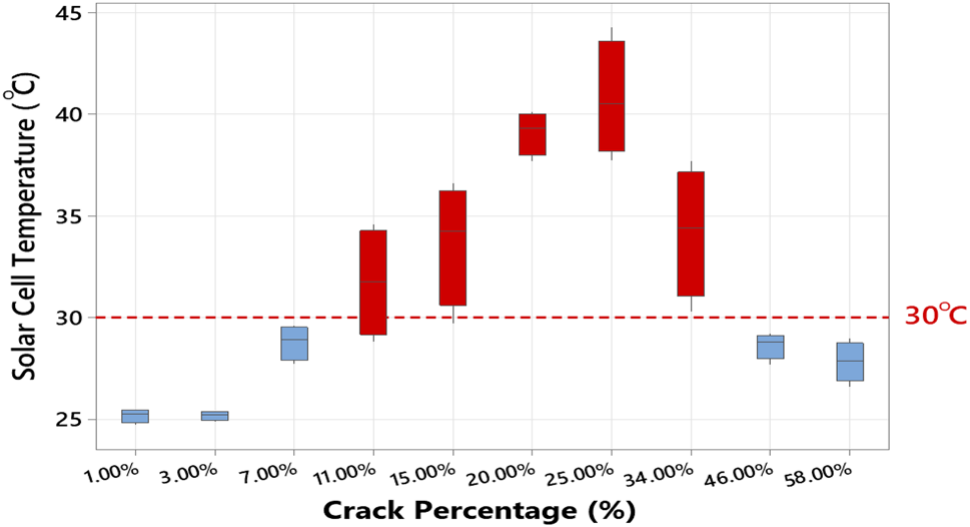
Solar cell samples temperature vs crack percentage.

The average surface temperature of the solar cells is then recorded using FLIR software and summarized in Fig. 7. It is recognized that a hotspot is to develop if the crack percentage, which is essentially representing an inactive area in the solar cell, is in the range between 11 and 34%. Considering the IEC61215/61646 standard, all other solar cells affected by either 7% crack size or below, or 45% crack size or more, are below the baseline of 30 °C; hence, these samples considered not correlate the crack and presence of hotspots.

Considering the results in Table 2, it is clear that the crack area has no increase in the temperature, always 25 °C or below. This is because the crack represents an inactive state. Hence, no absorption of electrons can be achieved, in other words, the inactive area has a temperature similar to the back or front sheet of the solar cell. Nevertheless, because of the uneven distribution of the current flow in the busbars due to the presence of the cracks, a localized heat (hotspot) can develop. For example, the surface temperature of the solar cell with a 20% crack percentage has increased to approximately 40 °C.

We have discovered that whenever the crack area increase, it is not necessary to develop a hotspot. A good example is given in Table 2 by the last two solar cell samples, 46% and 58%. Because there is a significant inactive area in the cells, the localized heat can be critical, yet there is not enough area to develop a hotspot. Therefore, in this condition, and as seen in Fig. 7, there is assuredly an increase in the temperature in some parts of the cell, yet the average cell surface temperature is below 30 °C.

**Figure 8:**
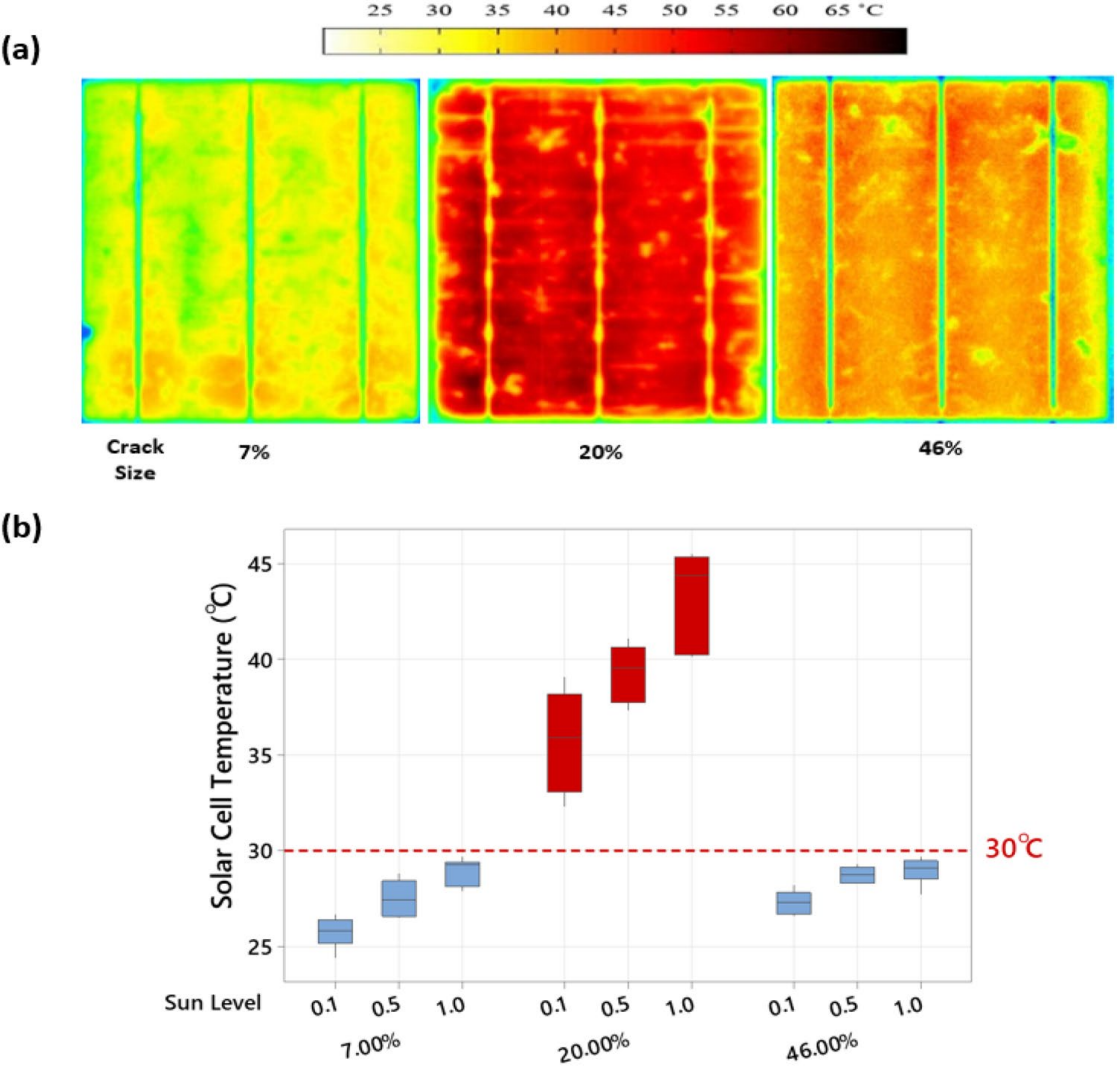
(**a**) Thermal image taken at the end of the experiment of the three tested solar cell samples, (**b**) Solar cell samples temperature vs Sun level.

To further explain this effect, we have considered experimenting with the solar cells' thermal cycle that is close enough to the baseline temperature, 30 °C. Therefore, both solar cell samples with crack percentages of 7% and 46% have been considered. And then compared with the thermal cycle of the solar cell that has a 20% crack percentage, which develops hotspots. Each cell was subjected to a change in the Sun levels (0.1, 0.5, 1, 0.1, 0.5 and 1), each Sun level (cycle) lasts for 1 min. The variations of the Sun levels ensure that the hotspot develops in the solar cells and certainly impacts the distribution of the heat. As shown in Fig. 8a, at the end of the experiment, the surface temperature has been increased of the three examined solar cell samples.

During the changes of the Sun levels, the average surface temperature was recorded and summarized in Fig. 8b. Here we can confirm no significant increase in the surface temperature across all Sun levels for the solar cells affected by crack percentages 7% and 46%. In contrast, the mean surface temperature is approximately 40 °C for the solar cell affected by a 20% crack percentage. This result verifies our previous remarks that the crack ratio is essential to be considered to explain whether hotspots is expected to develop in solar cells or not.

While understanding the results presented in this section, we realized that there might be a substantial similarity between the cracks and the original PID effect in output power losses and hotspots development. This correlation has been investigated, and the analysis is discussed in the next section.

### Solar cell power loss and hotspots development though PID testing

It is apprehended that PID is an utterly different degradation mechanism than cell cracking. PID results from a high voltage electric field and sodium Ion migration from the PV module glass to the cells^29^, while solar cell cracking occurs due to thermal and mechanical stresses^30^. Therefore, this section will present the output power losses of PID affected solar cells; the results will then be compared with the output power losses of the solar cell cracks discussed in the previous sections. This examination will demonstrate that solar cells' PID effect is not as severe as when cracked by 40% or more.

**Figure 9:**
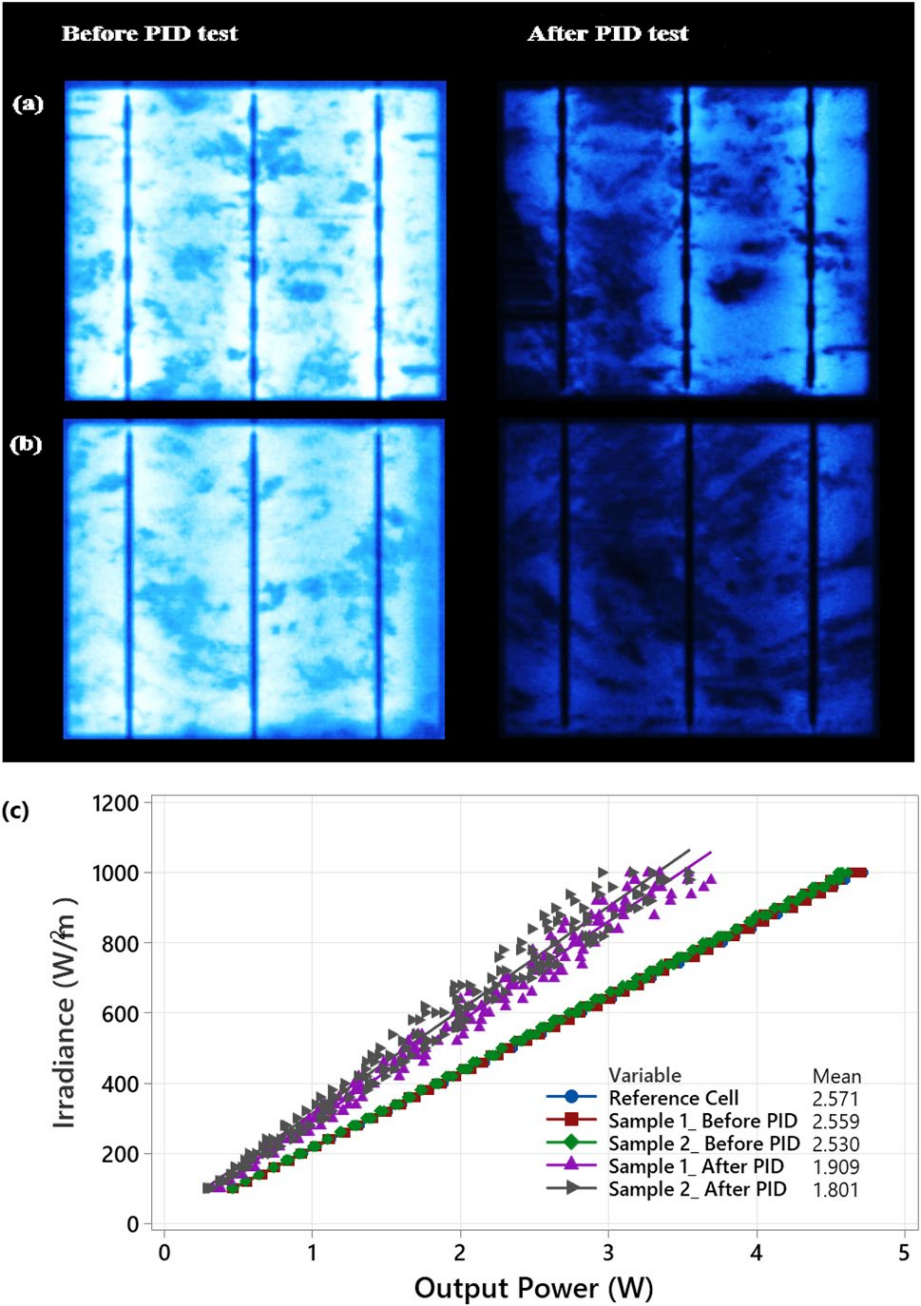
PID test output results (**a**) Sample #1, (**b**) Sample #2, (**c**) Output power measurements before and after the PID test was performed.

The PID test was performed using the PIDcon instrument. We have applied the standard PID testing conditions, where the temperature is maintained at 85 °C, the voltage is set to negative 1000 V, and the PID test ran over 96 h^28^. This procedure was performed on two solar cell samples that presents no cracks, shown in Fig. 9a,b. These figures also contain the EL image taken before and after performing the PID test. Apparently, the PID had a significant impact, as expected, toward developing shunted areas in the cells. Furthermore, the output power measurements for the solar cell samples before and after the PID test are presented in Fig. 9c. After the PID experiment was completed, it is seen that the mean output power has significantly dropped for both samples, 1.909 and 1.801 W.

**Figure 10:**
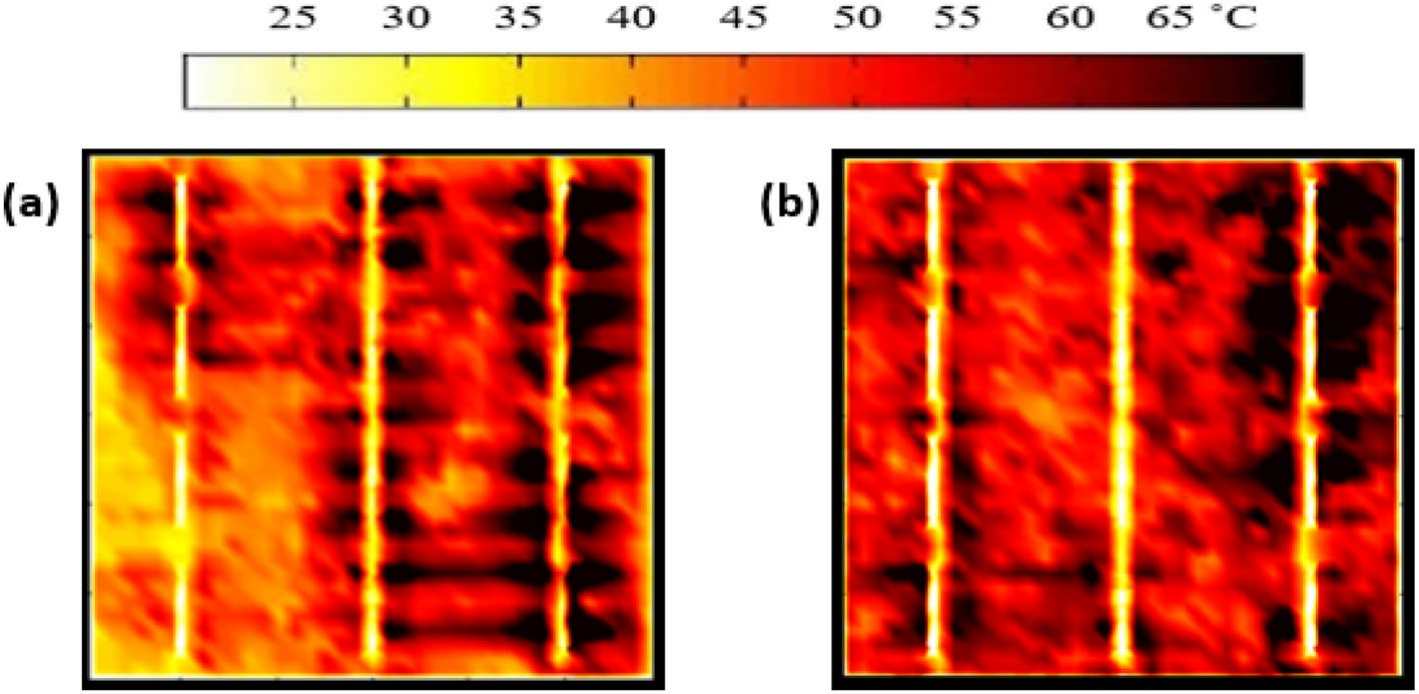
Thermal image of the solar cell samples after the PID test was completed (**a**) Sample #1, (**b**) Sample #2.

As we have shown earlier in Table 2, some solar cells do not develop hotspots. In this regard, the PID experiment allows us to confirm another compelling conclusion. We have observed that the solar cell samples subjected to the PID test have developed severe hotspots, as can be seen in Fig. 10. The surface temperature of both cells is approximately 50 °C; these images were taken at STC conditions. In comparison, the worst case of an increase in the cell temperature for cracked cells was observed at nearly 43 °C (Fig. 7) when the cell is affected by a 25% crack percentage. Therefore, unlike solar cracking, a PID can critically influence the thermal performance of affected solar cells.

**Figure 11:**
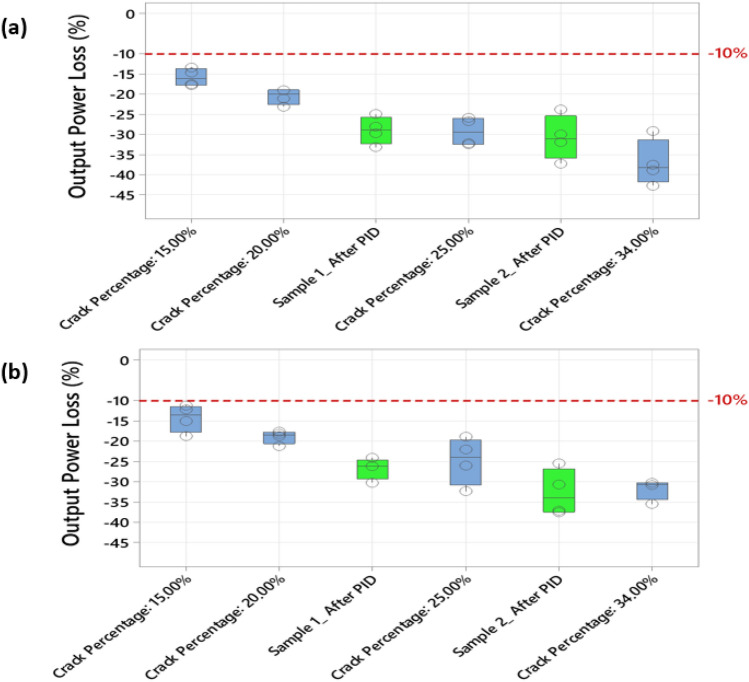
Calculation of the output power loss for the solar cell samples after PID test was completed, the results are also compared with the measurements taken from the cracked solar cell samples earlier shown in Fig. 6 (**a**) at 1 Sun, (**b**) at 0.5 Sun.

Furthermore, we compared the degree of similarity of the results obtained by the PID experiment against the cracked solar cell samples (Fig. 6). The outcome of this comparison is presented in Fig. 11. According to both Sun levels (1 and 0.5), that the solar cell samples subjected to the PID test are comparable to those with crack percentages of 20–34%. Consequently, the output power loss estimation ranges from − 20% to as low as − 35%. These results confirm to a certain degree that cracks in solar cells are a form of PID; they affect the output power performance and are unlikely to be mitigated. In summary, this section demonstrates that PID can rigorously impact solar cell performance, and cracks in solar cells are a form of PID. In the long run, both PID and solar cell cracks are likely to develop hotspots.

## Discussions

In this paper, we have presented the impact of solar cell cracks on their output power performance. Unlike some previous research work^3–5,21^, we have conducted experiments on various solar cells exposed to field conditions, obtaining an accurate estimation and analysis of the precise impact of cracks. Furthermore, we have also considered studying different solar cells affected by different crack sizes (1–58%), which is different from other recent research work^26,31^, which only considered studying PV module-level cracks (i.e., they did not investigate solar cell-level cracks vs crack sizes).

The thermal performance of all examined solar cell samples was also demonstrated. This experiment has indicated that not all cracks can develop hotspots because there is a substantial inactive area in the solar cell; thus, there is an insufficient area to establish a hotspot. For example, we have shown that when the solar cell is affected by crack size between 11 and 34%, it is likely to develop hotspots. This effect is usually ignored when examining solar cell cracks^31–33^.

Another contribution of this work is that we have presented the results of the output power degradation of two solar cell samples under the PID test. We have then correlated the power losses of the PID test results with the cracked solar cell samples. We have discovered that PID can result in 30% to 40% losses in the output power; this is pretty much the same amount of losses when a solar cell is affected by at least 25% cracks. Our results of the PID effect are similar to previous work^26,27^. However, the actual correlation between cracks, hotspots, and PID has not been yet investigated other than in this paper.

There are still some critical questions to be answered/linked with this research work; an example, how do cracks or hotspots develop over time? This has to be investigated over a long-term period for solar cells operating in diverse environmental conditions rather than simply examining the solar cells under artificial indoor illumination. In addition, we partially understand why/how hotspots' temperature changes over time (i.e., due to the difference in the summer-to-winter or day-to-night temperature^34,35^). However, further investigations must explain this critical problem affecting nearly most PV assets in today's PV market.

## Conclusions

We have studied the impact of cracks on the performance of solar cells investigated under different conditions. The findings are concluded as follows:

1. As the crack size increases in the solar cell, the output power loss increases. However, it was found that when the crack size is 1–7%, there are insignificant power losses for the affected solar cell samples.
2. We discovered that not all solar cell cracks develop hotspots. For example, no hotspots were found when a solar cell is affected by minimal (below 7%) or monumental (greater than 46%) crack sizes.
3. We have seen that the PID can degrade solar cells' output power by at least 20% after 96 h of the PID cycle. Compared with solar cell cracks, this is equivalent to a solar cell affected by at least 25% inactive area (crack size).

## Data Availability

The datasets generated and analysed during the current study are not publicly available due to the funding policy but are available from the corresponding author on reasonable request.
